# An integrate-and-fire mathematical model of sleep-wake neuronal networks in the developing mammal

**DOI:** 10.1371/journal.pone.0307851

**Published:** 2024-10-03

**Authors:** Adrian J. Samberg, Deena R. Schmidt

**Affiliations:** Department of Mathematics and Statistics, University of Nevada, Reno, Nevada, United States of America; Sorbonne Universite UFR de Biologie, FRANCE

## Abstract

Sleep behavior is present in nearly all animals, and is a vital part of growth, development, and overall health. Infant mammals cycle randomly between short bouts of sleep and wake, and the lengths of these bouts both follow an exponential distribution. As mammals mature into adulthood, the mean sleep and wake bout lengths increase, and we also observe a change in the distribution of wake bout lengths from exponential to power law. Focusing on three regions of the brainstem that are involved in sleep-wake regulation, we develop a novel integrate-and-fire neuronal network model to expand upon previous mathematical models of sleep-wake regulation in mammals, focusing on rats. This model allows fine control over neuronal connectivity while simultaneously increasing the size and complexity of the modeled system to make it more representative of reality. We establish a relationship between neuronal network structure and function that could explain the different sleep-wake behaviors observed in rats as they progress through development. We explore the relationship between three different neuronal populations as well as the overall network behavior of the system. We find that increasing synaptic connectivity strength between the wake-promoting region and the wake-active region accounts for the observed changes in mammalian sleep-wake patterns. This dynamic neuronal connectivity is a possible mechanism that accurately accounts for sleep-wake pattern changes observed during mammalian development.

## Introduction

Nearly all animals exhibit some sort of sleep behavior, and it serves important biological functions. Specifically in mammals, sleep is an imperative time for the body to create and solidify neural connections, repair tissues, and maintain homeostasis [1, 2]. Sleep cycles in mammals are well studied in terms of circadian rhythms, as well as how sleep patterns change throughout mammalian development. Infant mammals cycle rapidly between short bouts of sleep and wakefulness, and the length of such bouts both follow an exponential distribution [3–7]. As mammals mature into adulthood, the mean wake and sleep bout lengths increase, but we also observe a change in bout length distribution. Sleep bouts remain exponentially distributed while wake bouts transition to a power law distribution [3, 8–11]. For rats, postnatal day 2–8 (P2-P8) corresponds to the infant stage and P8-P21 represents development through adolescence to adulthood [3].

It is understood that in the mammalian brain, there are areas of the brainstem that are active during bouts of wakefulness (wake-active or WA populations), and other areas of the brainstem that are active during bouts of sleep (sleep-active or SA populations) [12]. To maintain sleep or wakefulness for any substantial duration of time, there is mutual inhibition of the WA and SA neuronal populations [5, 13]. This mutual inhibition creates a bistable stochastic system that can maintain either stable state for an extended period of time. Without any external stimuli present, the system would eventually settle into one state or the other permanently, so an external excitatory noise is necessary to initiate the switching behavior between states [14, 15]. Along with mutual inhibition, intrapopulation self-excitation helps contribute to the bistability of the system, and this is included in models that aim to replicate this behavior [14, 15]. Essentially, what has been established is a bistable neuronal system (the two distinct states being the WA state and the SA state) created by interpopulation mutual inhibition and intrapopulation self-excitation, with switching between states initiated by external noisy excitatory stimuli.

Being the most complex organ in the human body, the human brain is far too large and complicated to model all at once [16]. One approach to study sleep-wake behavior is to use a mathematical model to simulate a neuronal network with malleable connectivity. The objective, then, is to examine network behavior produced by models of proposed connectivity against data obtained from living animals. There is extensive research providing data about the sleep-wake behavior of rats from infancy through adulthood, however the physiological neuronal network changes that underlie this observed data remain largely unknown. Our goal is to provide a possible neuronal network with dynamic network changes over time that would create the observed behavior. One of the major brain regions associated with wakefulness (WA) is the dorsolateral pontine tegmentum and another region that corresponds with sleep (SA) is the nucleus pontis oralis [5]. Additionally, there is a third wake-promoting (WP) brain region thought to be responsible for the transition of wake bout distribution that occurs later in development, the locus coeruleus (LC) [17]. We focus on these three brain regions in our neuronal network model.

In this work, we develop a three-population (sleep-active, wake-active, and wake-promoting) neuronal network model of brainstem regions to replicate observed sleep-wake behavior in the developing mammal. We use an integrate-and-fire model which creates a framework with malleable connectivity. We have individual control over the synaptic strength within and between neuron populations. We also include an external excitatory noise that is incorporated independently for each neuron in the entire system, increasing both the complexity of the model and also how well it represents neuronal connectivity. Finally, we increase the neuron population size as compared to previous models.

We will confirm previously identified key aspects of the model to produce similar overall network behavior, including increasing WA-SA and SA-WA mutual inhibition to increase mean bout length during development, intrapopulation self-excitation that is weak compared to the interpopulation mutual inhibition to stabilize each separate state, and external noisy stimuli in the form of Poisson noise necessary to produce switching between the two states of a bistable stochastic system. We will observe the effects of this changing neuronal connectivity over time on the gross network behavior, specifically focusing on sleep and wake bout distributions. Finally, we will quantitatively investigate the interaction between the wake-promoting and wake-active neuronal populations, and the effect of this interaction on overall network behavior during different developmental stages. This includes concordance of WP activity and WA activity (how much WP activity changes during wake-activity vs. sleep-activity), WA mean bout length increase as WP-WA connectivity strengthens throughout development, and the shift in wake bout distribution from exponential to power law as the brain matures. We confirm that this dynamic neuronal network is capable of closely reproducing observed sleep-wake behavior, and this is a possible representation of neuronal connectivity in the mammalian brain. Our model is also well suited for expansion and is more flexible and scalable than previous models [18–20].

## Methods

### Background and previous models

**Fig 1:**
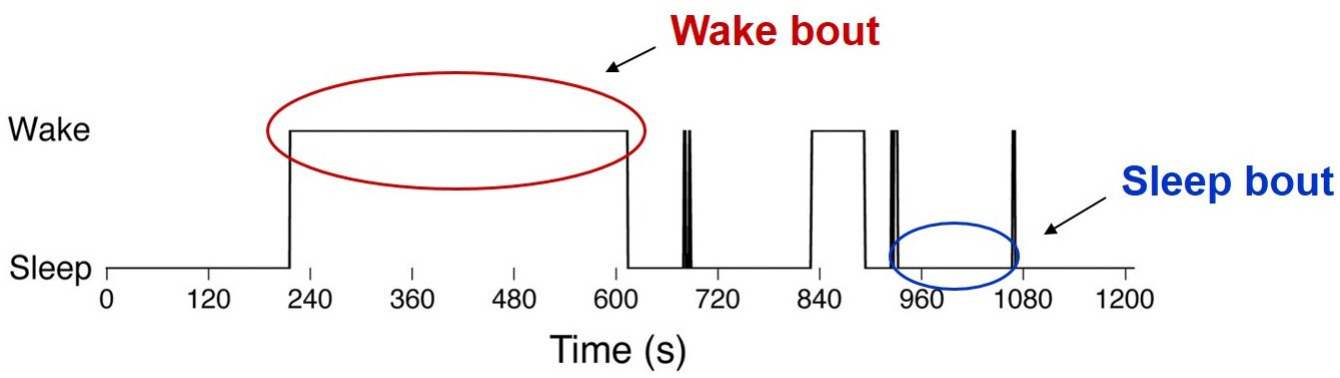
Rats stochastically switch between sleep and wake states. This figure shows a 20-minute electromyogram recording of an adult rat (modified from Fig 1 in [3]). A wake bout is the length of time spent in the wake state before transitioning to the sleep state. A sleep bout is the length of time spent in the sleep state before transitioning to the wake state.

Transitions from wake to sleep and sleep to wake are stochastic in mammals [9, 10]. Sleep occurs as a series of discrete bouts interrupted by brief awakenings (Fig 1). Several recent studies have analyzed the durations of sleep and wake periods (or bouts) in rats as well as other mammals, such as humans [3, 9]. For context, think of the sleep patterns of an infant human. Summed over an entire day, they may sleep for many hours, but this sleep occurs as a set of sleep bouts interrupted by periods (some brief) of wakefulness. Throughout development, this pattern stabilizes to more consistent and longer periods of sleep and wakefulness. We use data from rats (from infant to adult) as a goal for our model to replicate [3, 9, 10].

Previous models included smaller neuron populations, no wake promoting neuron population, or a different modeling framework to describe neuronal behavior [18–20]. We developed an integrate-and-fire neuronal network model to expand upon previous efforts [18].

It is important to understand some terminology before diving into further details. Neurons in the mammalian body are maintained at a resting membrane potential (RMP) of ≈ −70 *mV*. When the neuron’s membrane potential reaches a threshold (≈ −55 *mV*) a neuron ‘fires’, and an action potential (AP) or ‘spike’ is generated. The membrane potential increases rapidly to about +40 *mV* before returning to RMP. Membrane potential can be perturbed for a number of reasons. In terms of synaptic connectivity, a neuron generally receives either an excitatory or inhibitory connection. An excitatory postsynaptic potential (EPSP) will slightly increase membrane potential from RMP, and an inhibitory postsynaptic potential will slightly decrease membrane potential from RMP. Summation of all these excitatory and inhibitory connections to a specific neuron will determine its gross behavior. If after summing up all the EPSPs and IPSPs (+ and—connections), the membrane potential is above the threshold, an AP will be generated.

**Fig 2:**
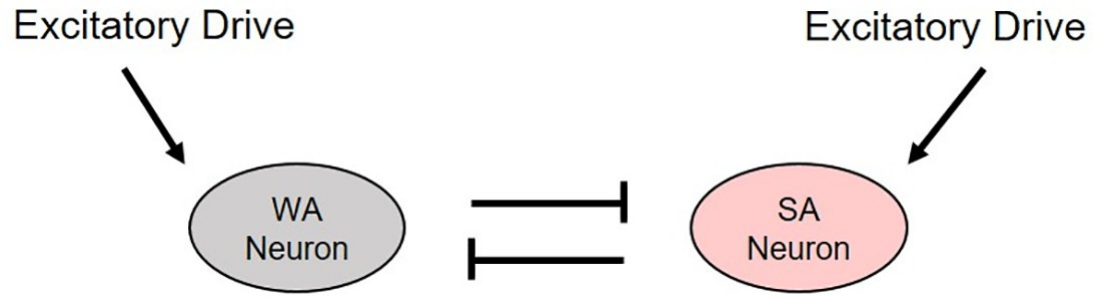
Model of one wake-active (WA) neuron and one sleep-active (SA) neuron. Mutual inhibition between the neurons sustains this basic bistable system, and excitatory drive in each population initiates switching behavior. This lays the foundation for further investigation into the dynamics of two mutually inhibitory integrate-and-fire neurons with independent noisy external excitatory drive to each neuron. This figure mimics Fig 1 of [19].

One of the most basic sleep-wake models uses an integrate-and-fire approach with two neuron populations with just one neuron each (Fig 2). In this model, both the wake-active (WA) neuron and sleep-active (SA) neuron have an independent noisy external excitatory stimulation which models spikes from outside the system, as well as inhibitory connectivity with the neuron of the other population (mutual-inhibition) [19]. This model is able to reproduce the general switching behavior observed in the sleep-wake cycles of infant rats. This is a necessary first step for modeling neuronal dynamics in sleep-wake systems, but this system is a vast oversimplification of the brain.

**Fig 3:**
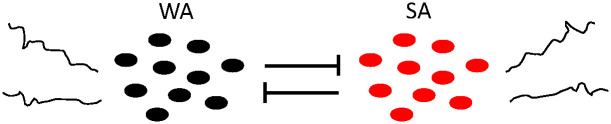
Model of two populations of ten integrate-and-fire neurons. This model has inhibitory all-to-all connectivity between populations and no within population coupling. Each neuron receives external excitatory spikes modeled as independent Poisson processes. This figure mimics Fig 1 of [20].

To increase the complexity of this system and make it closer to a real neuronal network, Patel [20] increased the number of neurons in each population to ten, connecting them via all-to-all inhibitory coupling and no within population coupling (Fig 3). This model confirmed that a noisy interference, representing neuronal signals from outside this network, is necessary to initiate the switching behavior. While this extra level of complexity does reveal important findings regarding the structure and function of the sleep-wake network in mammals, it only accounts for infant-stage observed behavior, and only includes the two basic neuronal populations: wake-active (black) and sleep-active (red).

**Fig 4:**
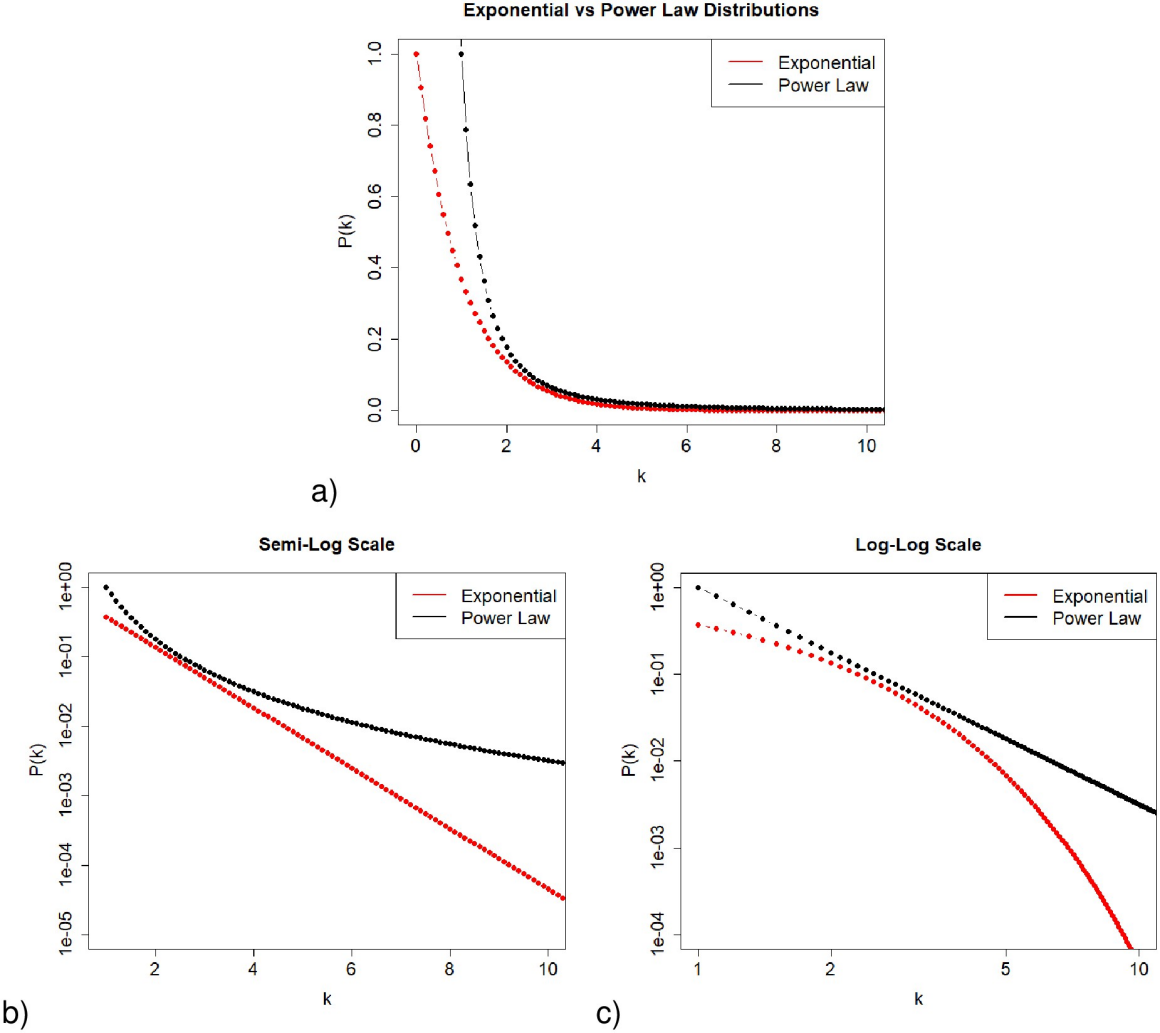
Comparison of power law and exponential distributions on different scales. a) Linear, b) Semi-Log, and c) Log-Log. Note that the exponential distribution is a straight line on a semi-log plot (panel b) and the power law distribution is a straight line on a log-log plot (panel c). The density function for the exponential distribution shown above has parameter *λ* = 1 so that *P*(*k*) = *λe*^−*λk*^ = *e*^−*k*^). The density function for the power law distribution is *P*(*k*) = *Ck*^−*α*^ = *k*^−2^ with parameters *C* = 1 and *α* = 2. See [22] for more about these distributions.

During the rat infant stage, postnatal day 2–8 (P2-P8), length of sleep and wake bouts both follow an exponential distribution, meaning short bouts occur more frequently than long bouts, appearing linear on a semi-logarithmic scale. During P8-P21, the length of wake bouts changes to a different distribution, known as a power law distribution, which is no longer linear when plotted on the semi-logarithmic scale, but is linear on a log-log scale (Fig 4). However, it’s important to note that, similar to a power-law distribution, a combination of exponential distributions can produce a linear relationship on a log-log scale [21].

**Fig 5:**
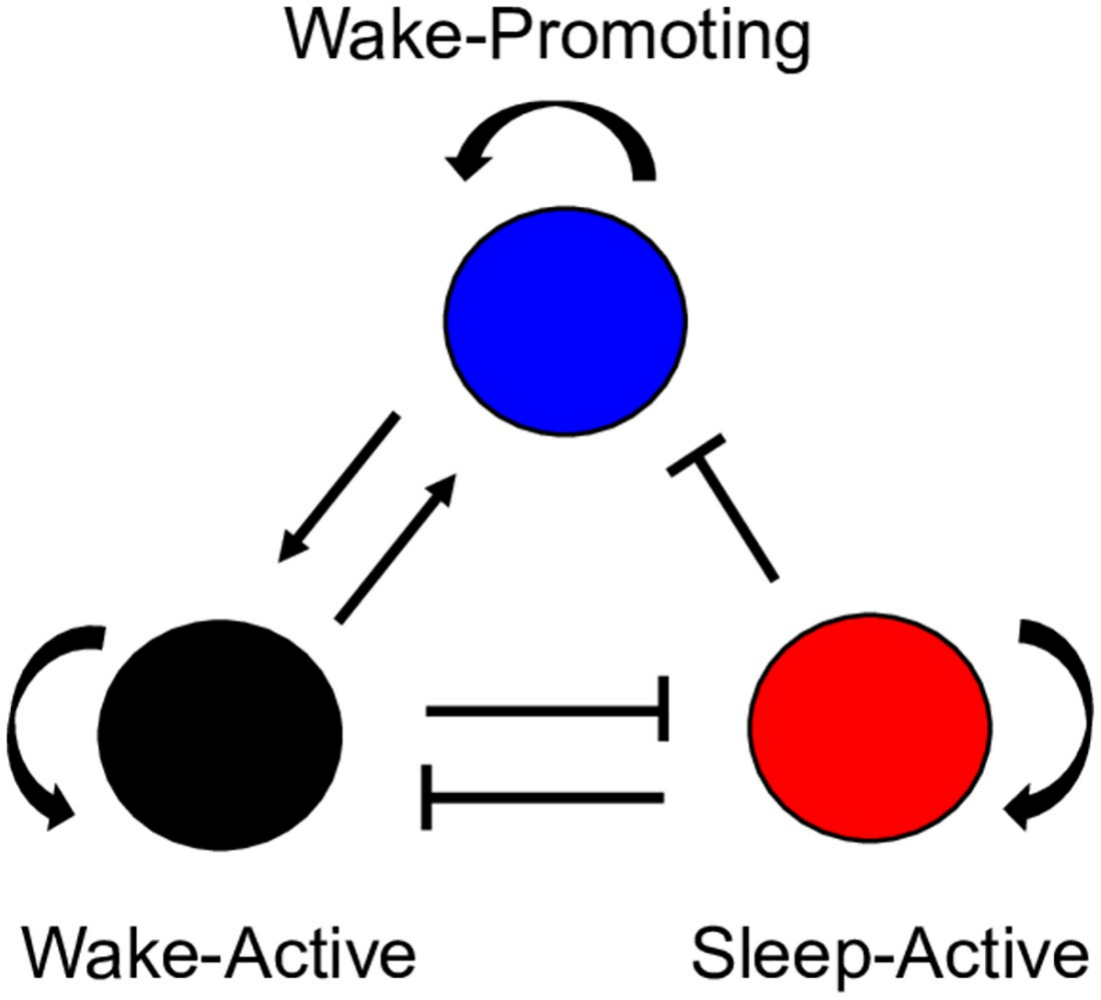
Model of three interacting populations of neurons. Patel and Rangan [18] integrated a third wake-promoting brain region to the infant stage neuronal connectivity using a Wilson-Cowan framework. The model includes three interacting populations of neurons that can account for the changes in sleep-wake behavior we see throughout rat development. This figure mimics Fig 1 in [18].

Further work aimed to understand this change in distribution and to develop a model that could apply to adult sleep-wake behavior. Changes in sleep-wake patterns during development are known to occur experimentally [3, 10], and the goal is to understand what underlying neuronal network changes accompany and contribute to this observed behavior. A third wake-promoting (WP) brain region is thought to be responsible for the observed shift in sleep-wake distributions throughout development [4]. This brain region, the Locus Coeruleus (LC), is a group of wake-promoting neurons known to undergo changes in firing patterns and connectivity with other sleep-wake brain regions during mammalian development. Lesioning the LC in the rat brain during infanthood prevents the transition of wake bouts to a power law distribution [4, 13, 23–25]. The change in LC interaction with the WA population coincides with the change from exponential to power law distribution, providing strong evidence that this network structure change is responsible for the transition [26–33]. The specific interactions of the LC and WA population in the mammalian brain are not well understood, but a proposed connectivity is shown below (Fig 5).

Fig 5 shows the model by Patel and Rangan [18], an expansion of the infant stage two population (WA and SA) model that includes the wake-promoting LC region. Addition of the LC into the sleep-wake neuronal network increases the complexity of the model of the developing rat brain to be more realistic, and helps gain insight as to the structural and functional connections of the LC and WA brain regions. Their model used a Wilson-Cowan style neuron model instead of an integrate-and-fire framework, and confirmed that mutual-inhibition had to be fairly strong in relation to the self-excitation of the WA and SA neuron populations [18]. We aim to further investigate the interaction between the LC (WP) and WA neuron populations that appear to be responsible for the transition of wake bout durations from exponential to power law distributed while sleep bout durations remain exponentially distributed.

### Integrate-and-fire neuronal network model

To continue this progression of work, we developed a new model that incorporates an integrate-and-fire framework, along with both interpopulation and intrapopulation neuronal connectivity. Importantly, we have the ability to tune the strength of LC-WA and WA-LC synaptic connections to emulate different stages of mammalian brain development. The model is flexible, allowing us to change parameter values for individual neurons if desired. We could draw a set of parameters for each neuron from a distribution instead of setting them all the same. There are many possibilities here.

**Fig 6:**
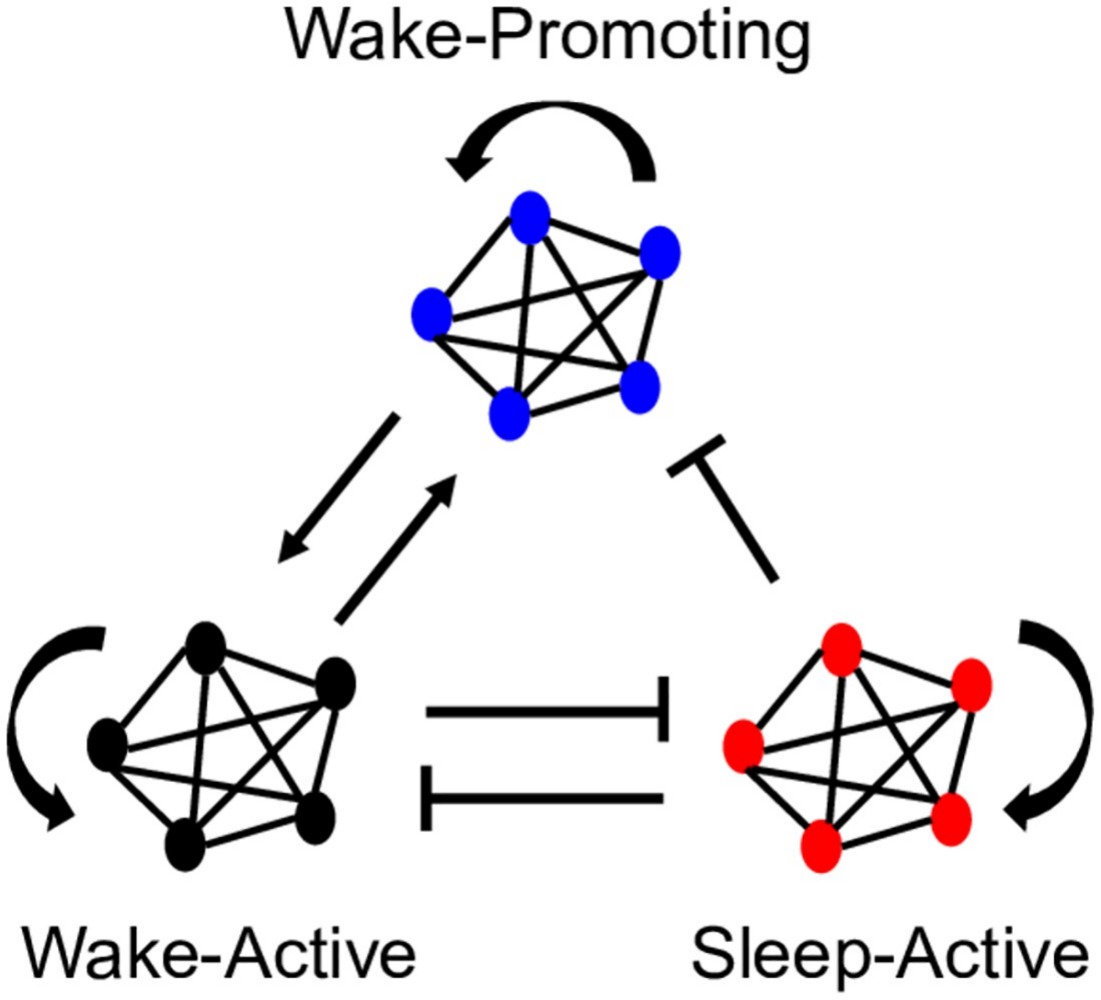
Neuronal network model including the wake-active, sleep-active, and wake-promoting brain regions. We assign N neurons to each population with all-to-all connectivity (this figure shows N = 5). A pointed arrow indicates an excitatory connection between brain regions, and a blunted arrow indicates an inhibitory signal. These arrows represent neuronal connectivity in the brain, and each neuron is modeled with a leaky integrate-and-fire differential equation.

We start with N = 5 neurons in each population to compare with previous work, and then we expand to larger N to confirm that our model scales to larger network sizes. Within each population, we assume all-to-all connectivity, meaning that all neurons within each population are connected to every other neuron in the population (Fig 6). This could easily be adapted to other types of intrapopulation connectivity. Synaptic connectivity that is crucial to proper sleep-wake behavior includes mutual inhibition between WA and SA, WA and WP mutual excitation, SA inhibition of WP, and self-excitation of WA, SA and WP (Fig 6). Strengths of synaptic connections are weighted differently (in both sign and magnitude) depending on the origin and destination. The change in strength of the different synaptic connections is malleable, and known to be vital to neural plasticity in living organisms. This turned out to be key to obtaining the desired network behavior from this model, particularly with tuning the strength of interaction between the LC and other neuron groups. Random noise was also introduced to each neuron population, and followed a Poisson distribution with parameter 1 with a multiplicative constant (p_WA_, p_SA_, p_WP_) for each population (see Table 3). Our random noise is an excitatory driving current delivered independently to each neuron, representing excitatory spikes from brain locations outside this network.

For our model to produce the desired behavior, we note that WA-SA and SA-WA mutual inhibition must be stronger than WA-WA and SA-SA self-excitation. We found that mutual-inhibition values must be approximately 2.5 times greater than self-excitation (Table 1). This is because, once in a bout, the neuron populations tend to stay in that state for an extended period of time before another switch occurs.

**Table 1:** Average sleep vs wake bout lengths as LC strength increases, N = 5 case.

LC Strength; N = 5 Case	Mean Sleep Bout (ms)	Mean Wake Bout (ms)
Infant: No LC	383.09	389.69
Adolescent: Moderate LC	279.55	1833.05
Adult: Strong LC	267.602	4305.98

We see that on average, sleep bout lengths remain similar throughout all trials, but average wake bout lengths increase dramatically as LC strength increases. This serves as a quantifiable measure of the effect the LC connectivity has on sleep and wake bouts and helps explain the visualized distribution changes.

Following the integrate-and-fire model, ordinary differential equations are used to model the change in voltage per unit time at each neuron in each population during each time step. We use one equation per neuron, and each equation depends on which population it is describing. In our case, each differential equation considers the membrane potential change due to leaky ion channels, how many spikes are received (both sign and magnitude), as well as random Poisson noise.

$$\frac{dv(t)}{dt}=I_{leak}(t)+I_{spikes}(t)+I_{Poisson}(t)$$

where

$$I_{leak}(t)=-\frac{C_{m}}{\tau_{m}}(v(t)-V_{0})$$

More specifically, for each neuron type, we have the following set of equations (Eq. 1):

$$\frac{dv_{WA}(t)}{dt}=-\frac{C_{WA}}{\tau }(v_{WA}(t)-V_{0})+(s_{WAWA}\;\frac{\Sigma_{WA}}{N_{WA}})+(s_{LCWA}\;\frac{\Sigma_{LC}}{N_{LC}})-{(s}_{SAWA}\;\frac{\Sigma_{SA}}{N_{SA}})+p_{WA}X$$

$$\frac{dv_{SA}(t)}{dt}=-\frac{C_{SA}}{\tau }(v_{SA}(t)-V_{0})+(s_{SASA}\;\frac{\Sigma_{SA}}{N_{SA}})-{(s}_{WASA}\;\frac{\Sigma_{WA}}{N_{WA}})+p_{SA}X$$

$$\frac{dv_{LC}(t)}{dt}=-\frac{C_{LC}}{\tau }(v_{LC}(t)-V_{0})+(s_{LCLC}\;\frac{\Sigma_{LC}}{N_{LC}})+(s_{WALC}\;\frac{\Sigma_{WA}}{N_{WA}})-(s_{SALC}\;\frac{\Sigma_{SA}}{N_{SA}})+p_{LC}X$$

These differential equations describe how each individual neuron’s voltage changes over time. I_leak_ is the current driving each neuron back toward resting membrane potential (RMP, approximately −70 *mV*) due to leaky ion channels. I_spikes_ is the current due to synaptic connections from which a neuron receives signals. As seen in Fig 6, a neuron may be receiving both excitatory and inhibitory signals simultaneously, but it is the sum of these values that sways the net effect on the neuron in question. I_Poisson_ is the random Poisson noise that changes at each time step. C_*i*_ is capacitance which relates to how quickly action potentials propagate (speed of a signal). *τ* is a timing constant that allows adjustment of how quickly a neuron will return to RMP after being perturbed. *v(t)* is the voltage at the current time step, and *V*_*0*_ is RMP. We denote the synaptic connectivity strength from one neuron population (*X*) to another (*Y*) as *s*_*XY*_. We use *Σ*_*X*_ to denote the sum of all spikes in population *X* during the current time step, and *N*_*X*_ is the number of neurons in population *X*. We also use a scaling parameter to make small adjustments in the integration of the Poisson excitatory drive, represented as *p*_*x*_ in the last term of each equation, and *X* is a random variable that follows a Poisson distribution with parameter 1. See Table 3 for exact parameter values.

**Fig 7:**
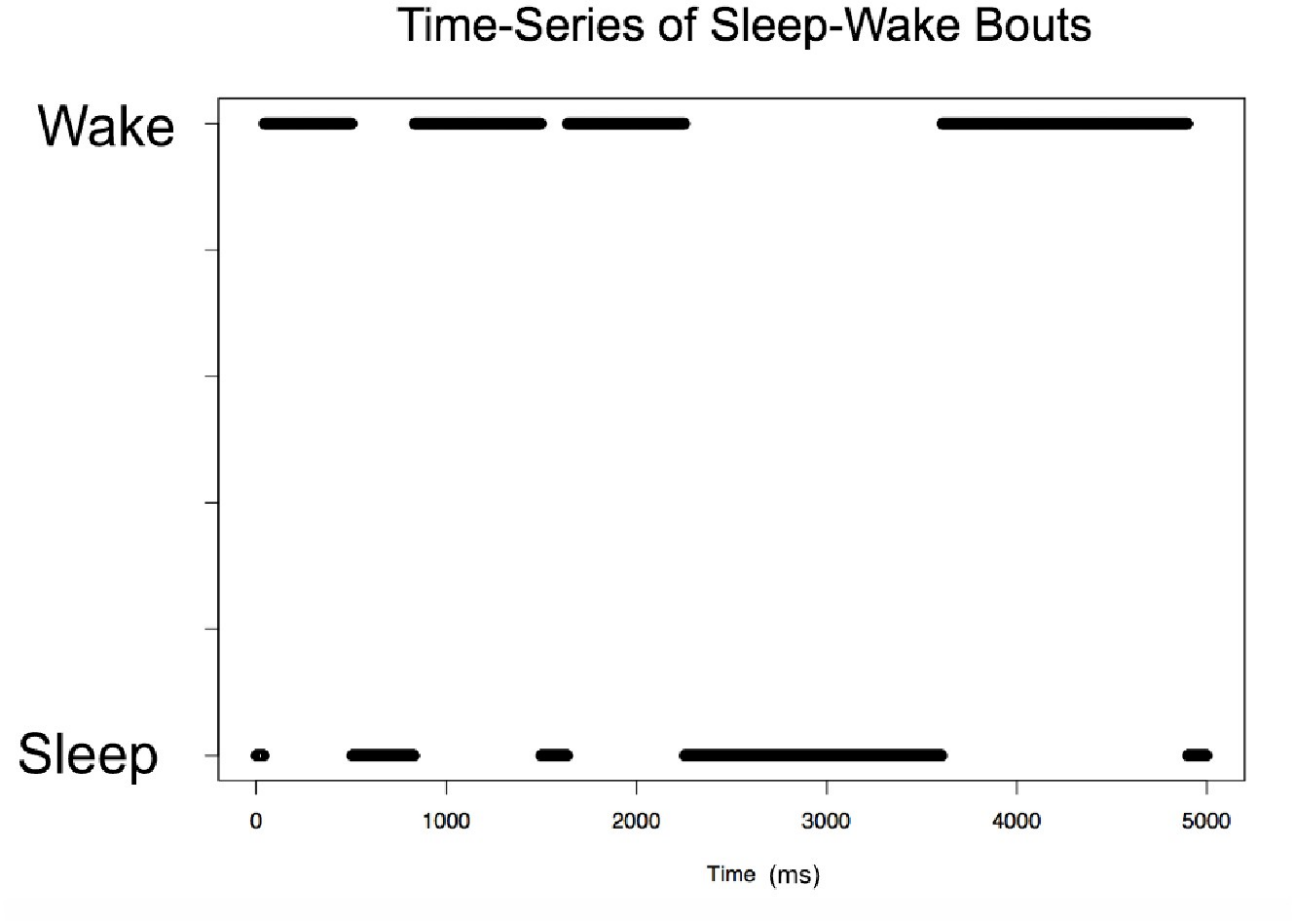
Switching between wake-active and sleep-active states. In this representative time frame (no LC connectivity, N = 5 case), we observe switches between WA and SA states in the model, as observed in real data. Time is measured in milliseconds. The population with more spikes during a 30 ms sliding window is defined to be the active population.

Due to leaky ion channels, the voltage of each neuron returns to its resting membrane potential once it is perturbed by an EPSP or IPSP. The spikes are action potentials that a neuron receives from neurons that are connected to it and perturb the voltage of the neuron to either hyperpolarize or depolarize it. Important to note is that these spikes are calculated as a sum; at any given time, a neuron may be receiving both excitatory and inhibitory signals, but the net effect on the neuron is the sum of all the spikes it is receiving (both positive and negative potentials). Finally, the random noise from the Poisson distribution is calculated at each time step for each neuron, and helps contribute as a stochastic variable to the switching behavior that is crucial for this model. This calculated membrane potential change is then summed with the potential at the previous time step, and evaluated for an action potential propagation (spike). As seen in Fig 7, we sum the total number of spikes for each population at every time step. This is used to determine which population is most active at any given time.

As an example, we discuss the WA population. As seen in Fig 6, each neuron in the WA population is receiving excitatory input from the WA population, excitation from the WP population, and inhibition from the SA population. During any given time step, a neuron will have its voltage perturbed by the current from multiple locations. Due to leaky ion channels present in the cell membrane of neurons, if the voltage of the neuron is different from RMP, voltage will be altered slightly in the direction toward RMP. Additionally, the neuron will be summing the weighted currents from each of its neural connections. The weights of these connections are the synaptic strengths, which are the variables we are changing to represent the mammalian brain in different stages of development. By changing the synaptic strength, we are representing the proposed change in neuronal structure that takes place as the brain develops that accounts for the observed sleep-wake behavioral changes.

### Results for small network model: N = 5 neurons in each population

We successfully maintain the desired switching behavior between sleep and wake bouts in our model, lending to maintenance of the stochastic bistable system (Fig 7). Sleep and wake state are defined by which neuron population has more spikes (APs) at any given time using a sliding window of 30ms. Simulations of our model for the infant stage (the case of no LC connectivity) show discrete bouts of sleep interrupted by periods of wakefulness. This figure only shows a small portion of time (5000 ms), so the arousal state changes are visible. It is important to note the variation in bout lengths for both sleep and wake states. We use the data from this plot over long simulations to gather information of sleep and wake bout lengths, and use that information to generate distributions.

We confirm that changing the mutual inhibition strength between the WA and SA populations changes the mean bout length by conducting trials where only these two variables were changed (Table 2). We see that small changes in the connectivity strength regarding mutual inhibition can independently and significantly have a large effect on bout length for both WA and SA simultaneously. This is an important characteristic of the neuronal network model as one key finding from experimental research is the overall increase in mean bout length for both sleep and wake. In terms of variability, we also looked at the standard deviation of sleep and wake bout lengths and note that these range from 1.4 to 1.7 times higher than the mean values.

**Table 2:** Mean bout lengths under different mutual inhibition strengths.

Mutual Inhibition Strength; N = 5 Case	Mean sleep bout length (ms)	Mean wake bout length (ms)
Weak (75% strength)	154.78	147.44
Moderate (100% strength)	393.57	382.21
Strong (125% strength)	784.86	794.84

By simply changing the strength of the mutual inhibition between WA and SA neuron populations, we can change the mean bout length. Increasing mutual inhibitory synaptic strength increases both the mean wake and sleep bout lengths, and decreasing the mutual inhibitory synaptic strength decreases both the mean wake and sleep bout lengths. See Table 3 for further details of how these synaptic strengths compare to other synaptic connectivity values.

**Table 3:** Parameter values for small network model (N = 5 cases) representing various stages of development.

Parameter Values	No LC-WA connectivity	Moderate LC-WA connectivity	Strong LC-WA connectivity
*s*_*LCWA*_	0	3	4
*s*_*LCLC*_	0	2	2
*s*_*SAWA*_	24	24	24
*s*_*SALC*_	0	2	3
*s*_*SASA*_	6	6	6
*s*_*WAWA*_	6	6	6
*s*_*WALC*_	0	2	3
*s*_*WASA*_	24	24	24
RMP	-70	-70	-70
Threshold	-55	-55	-55
*C*_*i*_	1.0	1.0	1.0
*τ*	15.0	15.0	15.0
*p*_*WA*_	1.05	1.05	1.05
*p*_*SA*_	1.05	1.08	1.08
*p*_*WP*_	1.0	1.0	1.0

Parameter *s*_*XY*_ refers to the directed synaptic connection from population *X* to population *Y*. Note that all synaptic connection strength values are positive in this table because excitatory and inhibitory signs are taken into account in the differential equations (Eq. 1). Also, *C*_*i*_ could be different for each population *i* in {WA, SA, WP}.

**Fig 8:**
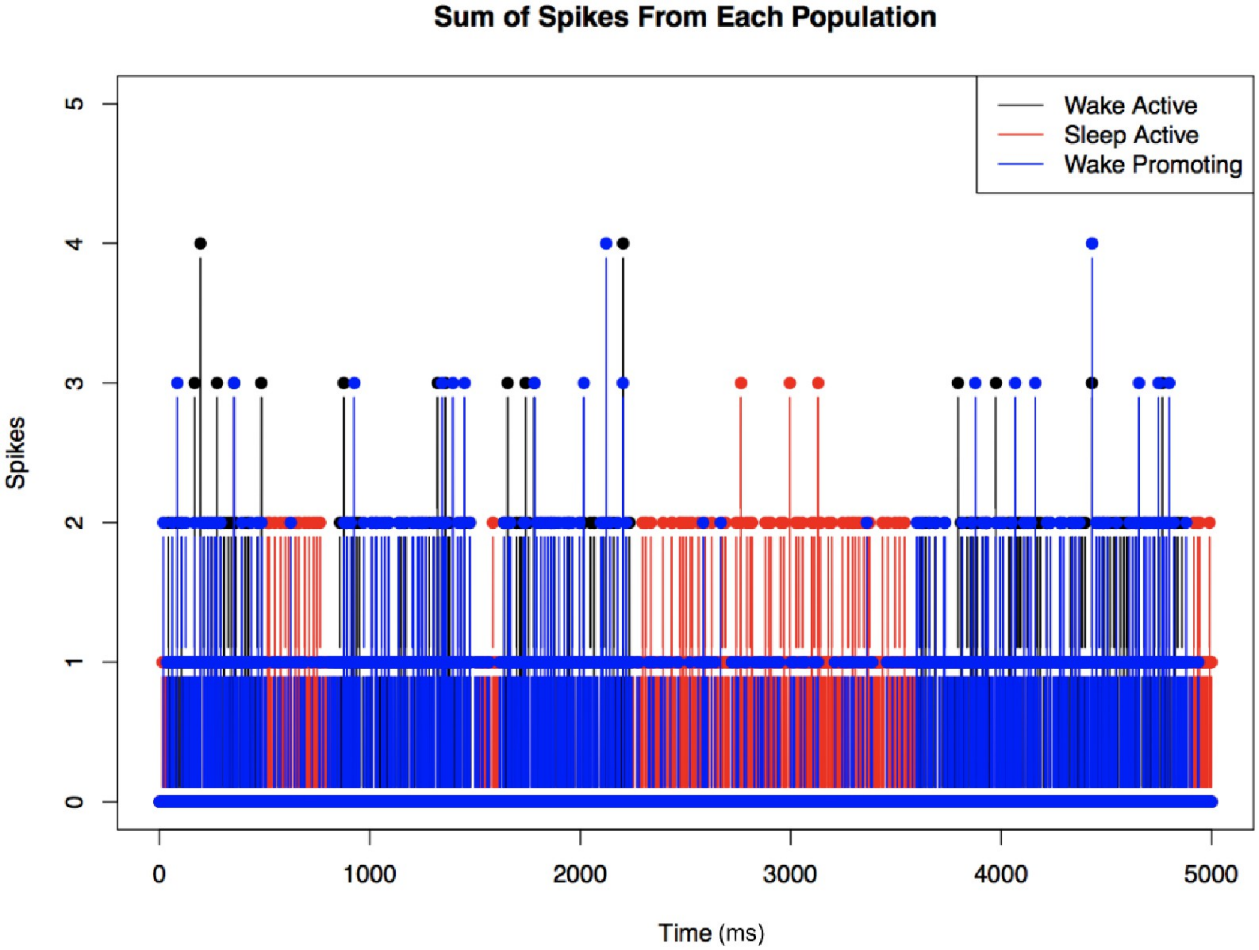
Another look at neuronal network switching behavior. Switching behavior observed for the moderate LC connectivity case (see Table 3) as summing the total number of action potentials/spikes in WA, SA, and WP populations; N = 5 case. More spikes in a certain neuron population correlate with higher neuronal activity in that population. Note that blue (WP) and black (WA) spikes tend to overlap, and areas of SA activation appear red.

One way to visualize the activity of the different neuron populations over time is to quantify the number of spikes (action potentials) in each population at any given time. Fig 8 shows the case of moderate LC connectivity (see Table 3 for parameter values). We note here that WA (black) and WP (blue) neuron populations tend to have correlated activity, as we’d expect. SA is red, and we see less WP activity during a SA bout. We can see that the degree of activity varies over time, and there is a clear separation between sleep and wakefulness.

**Fig 9:**
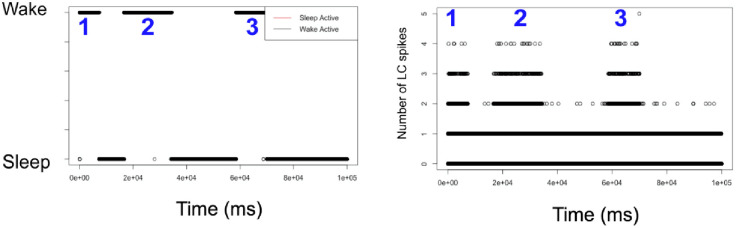
Correlative activity of the wake-active and wake-promoting LC populations. Moderate LC connectivity (see Table 3), N = 5 case. **Left:** Sleep and wake bout switching behavior with areas 1, 2, and 3 representing three sequential wake bouts. **Right:** Sum of LC spikes over time. In this example, we see three clear WA bouts during which there is a correlated increase in the number of LC spikes during a WA bout.

Fig 9 shows a further visualization of the correlative activity of the LC and wake bouts for the case of moderate LC connectivity (see Table 3). Panel a) shows a familiar plot of wake and sleep bout switching behavior. Panel b) shows the sum of the LC spikes for each time step. Comparing a) and b), we observe an increase in LC activity correlating with WA bouts and a decrease in LC activity during SA bouts.

**Fig 10:**
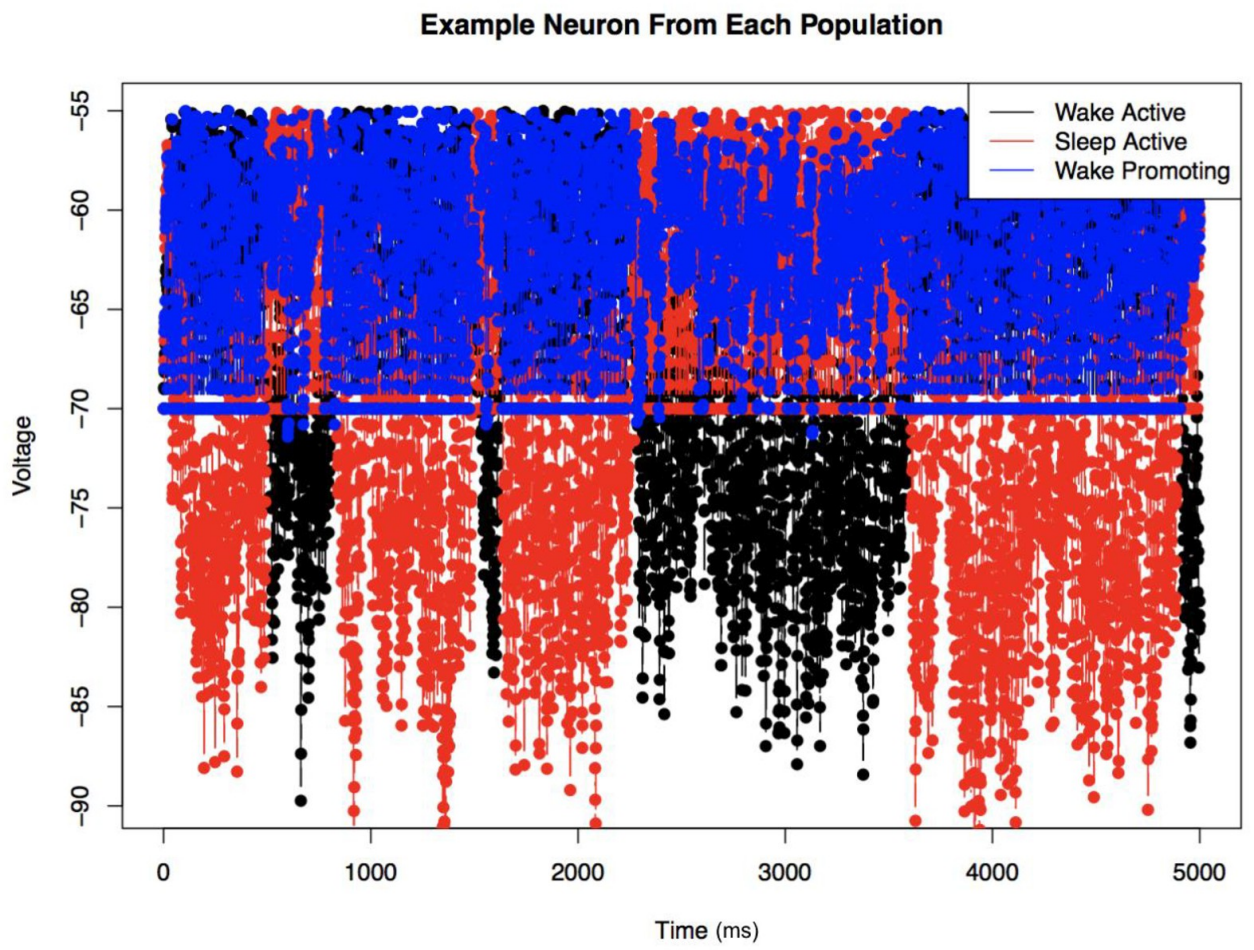
Voltage in a representative neuron from each population over time. This shows the case of moderate LC connectivity (see Table 3 for parameter values, N = 5 case). This allows us to observe the switching behavior, but also the change in activity in the WP region during the different arousal states.

We can see this process in a bit more detail in Fig 10. By visualizing the voltage of a representative neuron from each of the populations over time (moderate LC connectivity; see Table 3), we can more directly observe the mechanisms by which this model produces the expected behavior. Initially, we can discern the stochastic switches between arousal states of wakefulness and sleep. Here, however, we can observe the effects of strong mutual inhibition between the sleep- and wake-active regions. During a wake bout, the wake-active population has a depolarized voltage consistently above RMP, while the sleep-active population has voltage pulled even further hyperpolarized, consistently below RMP. This connectivity creates a large difference in the voltage between the two neuron populations, leading to the network holding in a certain arousal state for an extended period of time. The switching occurs when a large enough random noise can overcome this difference and a switch from WA to SA or SA to WA occurs.

Also important to note from Fig 10 is the change in activity of the WP neuron population during the different arousal states. Because of the network structure, we see an increase in activity of the wake-promoting region during a wake bout. Essentially, these two neuron populations are active simultaneously and work together as a positive feedback loop, creating that extra stability in the wake-active state. This figure allows us to observe the interaction and effects of the WP region on network behavior.

**Fig 11:**
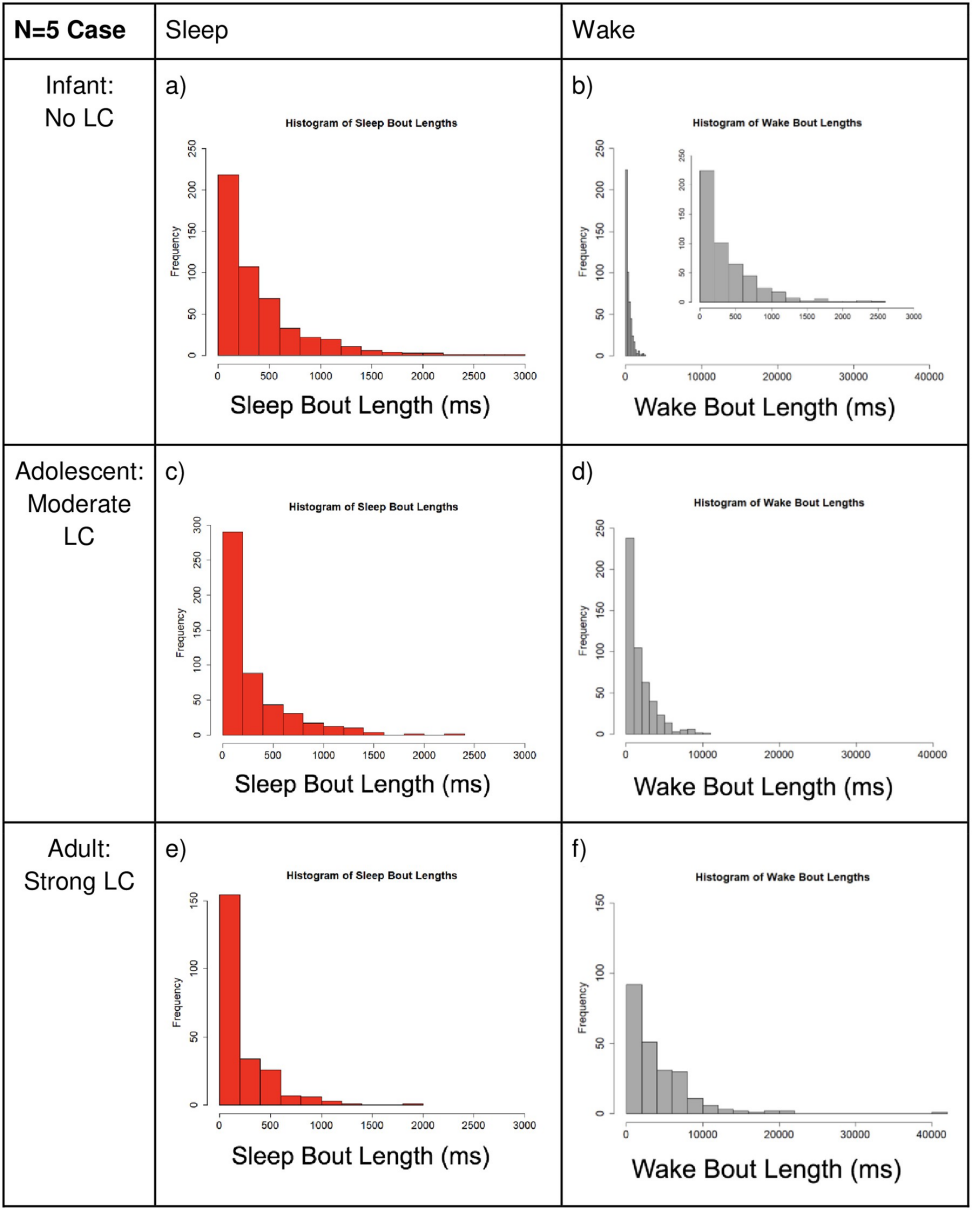
Small network comparison of the distribution of sleep and wake bout lengths. Small network refers to the N = 5 case, and the level of LC connectivity increases as we move down the rows. Note the y-axis varies slightly between plots, but the x-axis stays consistent per neuron population for all cases. **Left (SA case):** We see that increasing the LC strength does not change the average bout length much, and the distribution maintains the original exponential shape. **Right (WA case):** Wake bout lengths get much longer on average as LC strength increases, and the shape of the distribution of bout lengths differs noticeably from the original exponential shape (no LC interaction). Note that the inlaid graph in panel b), we include a plot of the same data with the x-axis matching the SA case; this demonstrates that with no LC interaction, the WA and SA distributions are nearly identical. Large x-axis is used for WA plots to show the large increase in observed bout lengths as LC-WA interaction is increased.

Fig 11 illustrates the distributions of sleep and wake bouts. We observe that the distribution of wake bout lengths changes as the locus coeruleus (WP) neuron population synaptic strength increases, which is what we’d expect (see Table 3 for exact changes in WA-LC and LC-WA connectivity). As seen in Fig 11(a), 11(c) and 11(e), the sleep bout distribution remains nearly identical despite LC synaptic strength increasing. Per real world data, we did not expect the sleep bout distribution to change. In panels b), d), and f), the wake bout distribution changes dramatically as LC strength is increased. Note the difference in x-axis scale between the wake and sleep columns. This new distribution has a heavy tail, meaning we see more long WA bouts and less short WA bouts, which is consistent with a transition to a power law-like distribution.

Below, we include a table to show how the mean bout length is affected by the change in LC-WA interactions. Again, reference Table 3 for exact changes in parameter values. This shows us that change in the LC synaptic connectivity does also contribute to the increase in mean bout length for the WA population. The heavy tail of the new distribution has a large effect on the mean bout length.

**Fig 12:**
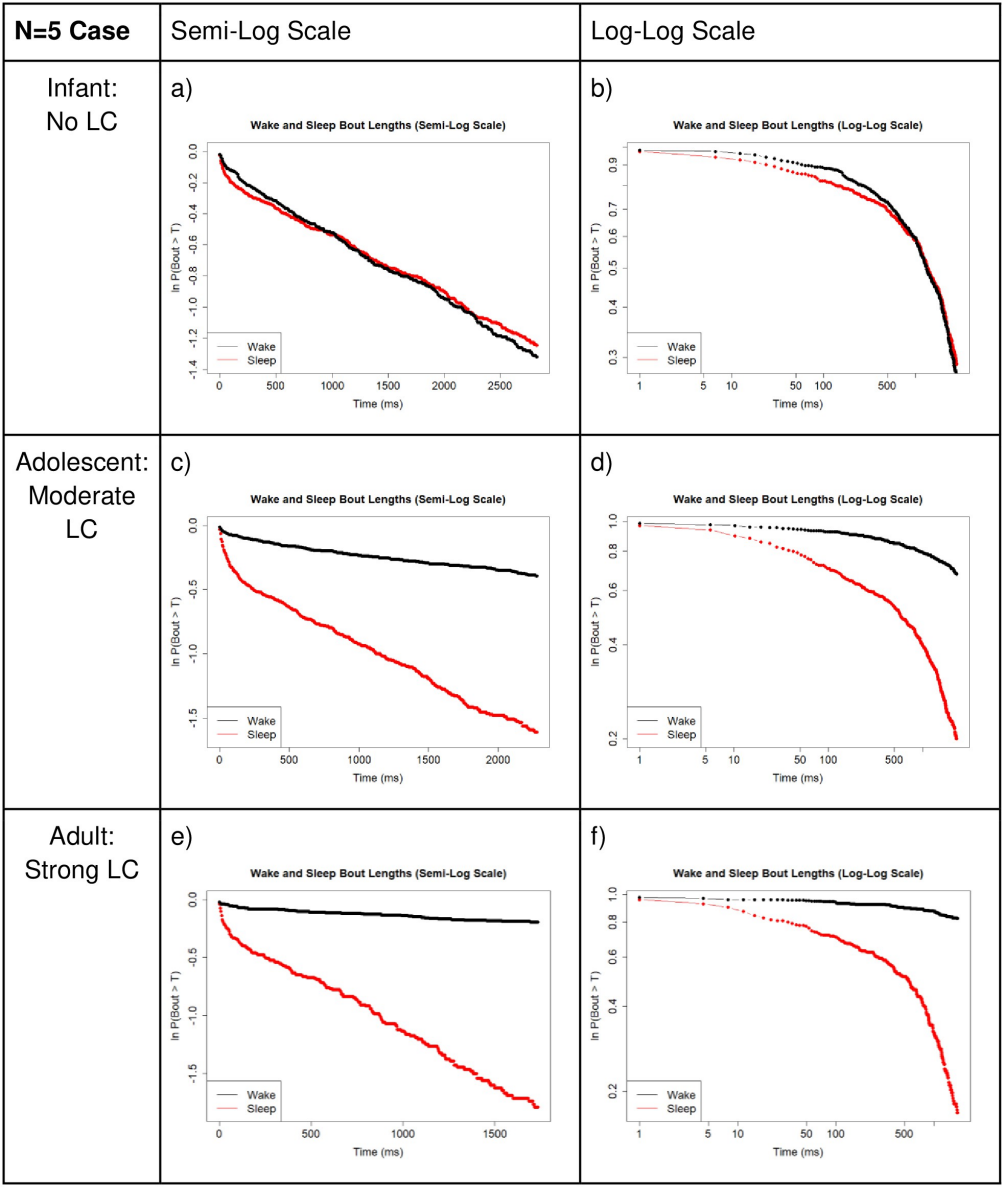
Small network comparison of survivor plots. Comparison of survivor plots of wake and sleep bout length distributions as connectivity strength between the WA population and LC population is increased, N = 5 case. **Left:** Semi-log scale; **Right:** Log-log scale. We observe the predicted shift in wake bout distribution from exponential to power law-like once the model brain has reached adulthood. Compare to Fig 4, which shows that a power law distribution is linear on a log-log scale. As we progress from panels b) to d) to f), we observe the wake bout distribution becoming more and more linear as these connectivity strengths are increased (see Table 3 for notable changes in *s*_*LCWA*_, *s*_*WALC*_, *s*_*SALC*_, and *s*_*LCLC*_). Also, as we’d expect, in panels a), c), and e), we note that the sleep bout distribution stays similarly shaped (nearly linear) on a semi-log scale, which as noted in Fig 4, denotes maintenance of an exponential distribution. These data were obtained from 1,000,000 time step simulations.

In Fig 12, we compare sleep and wake bout length distributions over varying degrees of synaptic strength between the LC and WA neuron populations on semi-log and log-log scales. We see that with no LC presence (infant stage), there are nearly identical distributions of SA and WA bout lengths (Fig 12a and 12b). Also note that both SA and WA bouts are essentially linear on the semi-log plot (Fig 12a), indicating an exponential distribution (Fig 4). As LC synaptic strength is increased to a moderate value (see Table 3), representing an adolescent rat, we see a shift to more long WA bouts and a minimal change in SA bouts (Fig 12c and 12d). Finally, as LC-WA synaptic strength is increased to a large value, signifying rat adulthood, we see a large difference in bout distributions, with a majority of long WA bouts and still relatively no change in SA bouts (Fig 12e and 12f). See Table 3 for specific parameter values. Importantly, note that the WA bout distribution is becoming linear on the log-log plot (Fig 12f), indicating the transition to a heavy-tailed distribution consistent with a power law-like distribution (Fig 4).

Another quantitative analysis carried out was to compare the LC firing rate during a sleep bout versus a wake bout. Based on the neuronal connectivity, we would expect a change in LC firing rate to correlate with a change from sleep to wake and wake to sleep. It only makes sense to consider this comparison when there is LC interaction, so we will focus on the strong LC case. Taken from a representative 1000 ms time step sample, the total number of LC spikes was found to be 227 LC spikes during a WA bout, and 85 LC spikes during a SA bout. This equates to 227 spikes per second for the entire LC population during a wake bout, or 45.4 spikes per second per neuron. During a sleep bout, we note that this rate drops to 85 spikes per second for the entire LC population, or 17 spikes per second per neuron. This corresponds to 2.67 times more activity in the LC brain region during a wake bout compared to a sleep bout.

### Results for larger network model: N = 500 neurons in each population

Here we expand our model to a larger network of neurons and show that our results are replicable at a more realistic neuronal population size. We increased our network size 100-fold from N = 5 to N = 500 neurons per population, meaning that our three-population model now has 1500 total leaky integrate-and-fire neurons. We analyzed this version of the model and created similar plots to compare to the N = 5 case, and we found similar results as described below.

**Fig 13:**
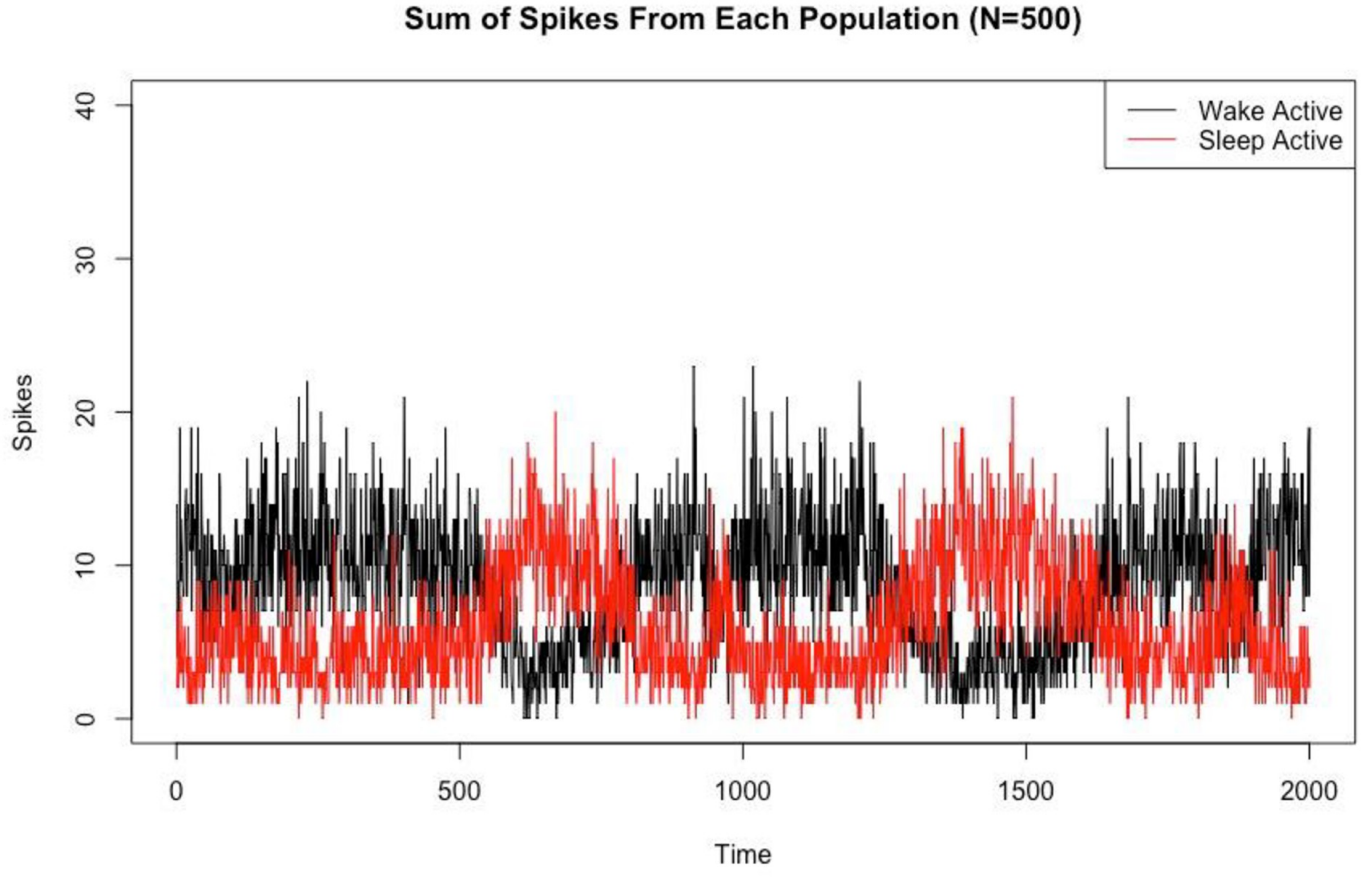
Switching behavior in the larger network model. Sum of spikes from each population for the Strong LC case for N = 500 (we omit the wake promoting population for clarity). This allows us to observe the switching behavior between sleep and wake states. We calculate which behavioral state the system is in by using a 30ms sliding window and counting which population (WA or SA) has the larger number of spikes. Time is measured in milliseconds.

As in the N = 5 case, Fig 13 compares the total spikes from the WA and SA populations within a 30 ms sliding window to define which population is active over time for the case N = 500. We see that in this larger network, we still have clear switching behavior between wake and sleep states, and the overall network behavior maintains its bistability.

**Fig 14:**
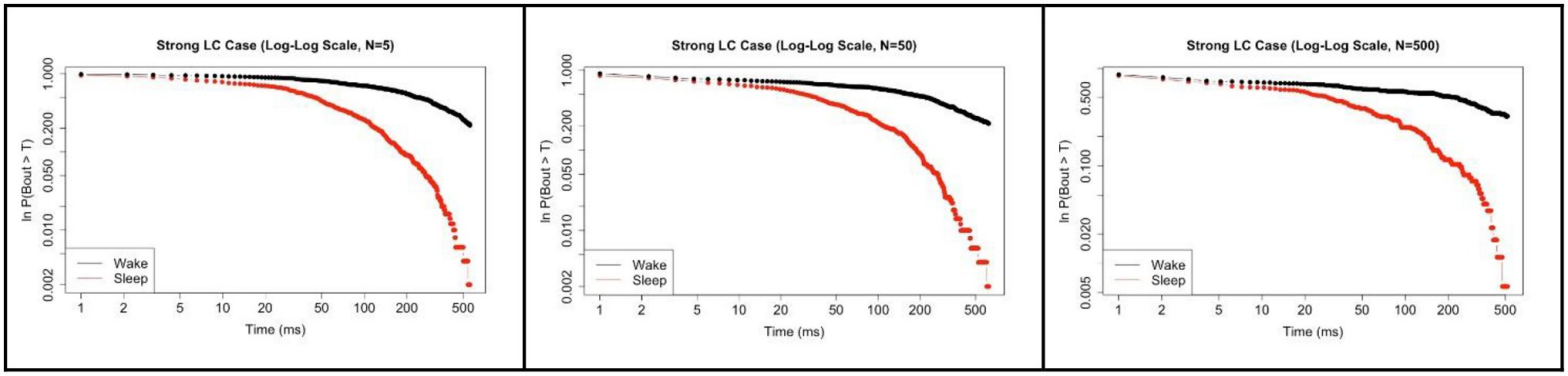
Comparison across increasing network sizes. Comparison of survivor plots of wake bout and sleep bout distributions (log-log scale, strong LC case only) as the network size is increased: N = 5 (**left**), N = 50 (**middle**), and N = 500 (**right**). Under strong LC interaction conditions, we see maintenance of the exponential distribution for the SA bouts, as well as the transition from exponential distribution to a power-law like distribution for the WA bouts. These plots were generated using 500K time steps for the N = 5 case, 200K time steps for the N = 50 case, and 100K time steps for the N = 500 case.

In Fig 14, we show that our model works well for both N = 5 and N = 500, and it is appropriate for any value one chooses in between. We show N = 50 as an intermediate case. The flexibility of our model is many-fold and well suited for further work and analysis. In stepping up to the larger size (N = 500), we found that we did need to alter the values of the synaptic connectivity parameters to maintain network behavior. Importantly, however, we want to emphasize that although the parameter values changed, the No LC, Moderate LC, and Strong LC cases were altered in an identical fashion to the N = 5 case. See Table 4 for the synaptic strength parameter values in the N = 500 case.

**Table 4:** Synaptic strength parameter values for larger network model (N = 500 cases).

Parameters	No LC	Moderate LC	Strong LC	WA Increase
*s*_*WAWA*_	0.8	0.8	0.8	1.1
*s*_*WASA*_	17	17	17	17
*s*_*WALC*_	0	0.1	0.2	0
*s*_*SASA*_	0.8	0.8	0.8	0.8
*s*_*SAWA*_	17	17	17	17
*s*_*SALC*_	0	0.1	0.2	0
*s*_*LCLC*_	0	0.05	0.1	0
*s*_*LCWA*_	0	0.1	0.2	0
*s*_*LCSA*_	0	0	0	0

Parameter *s*_*XY*_ refers to the directed synaptic connection from population *X* to population *Y*. Recall that LC synaptic connectivity is increased in multiple neuron population interactions in order to produce desired gross network behavior.

**Fig 15:**
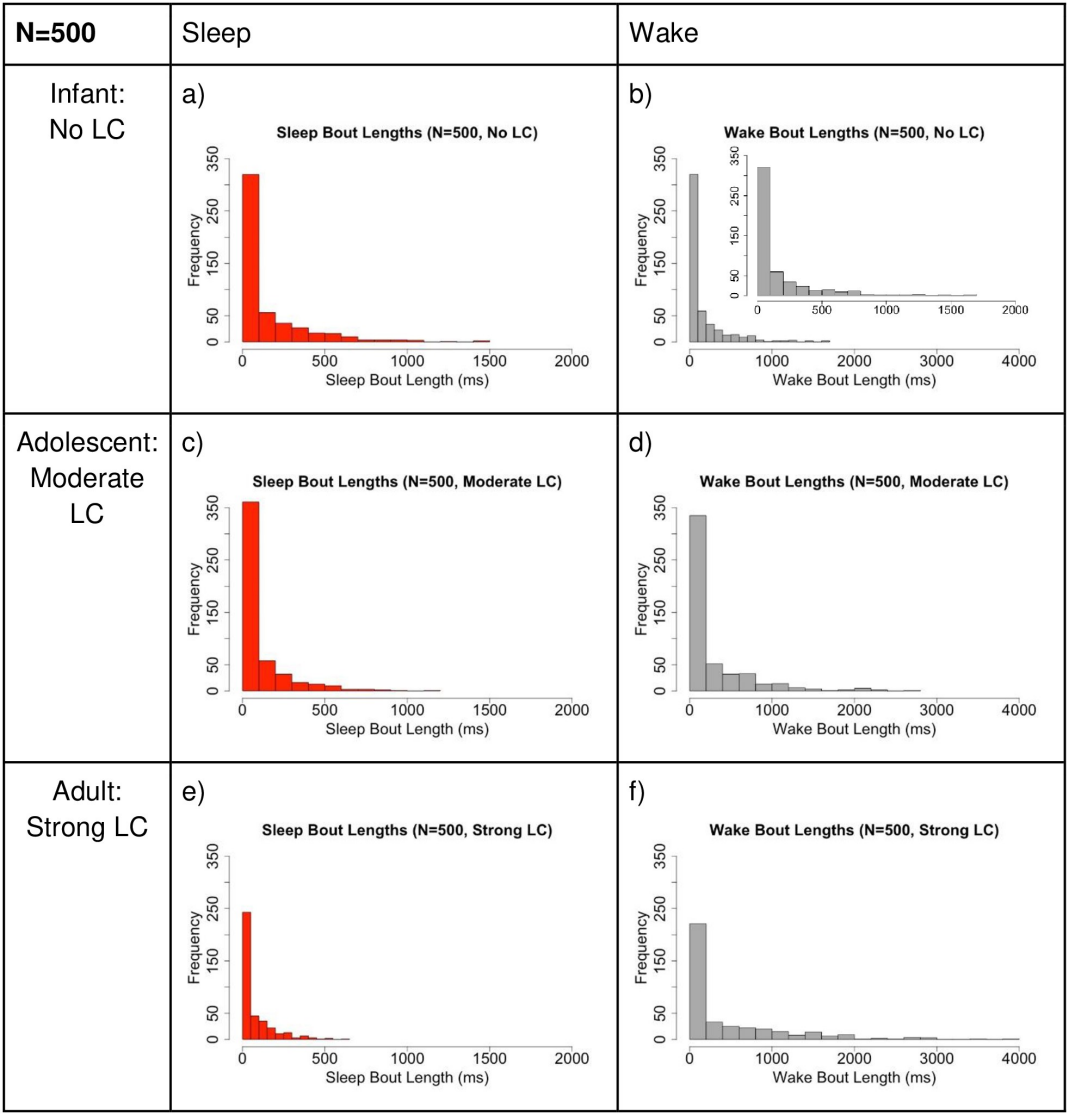
Larger network comparison of the distribution of sleep and wake bout lengths. Comparison of the distribution of sleep vs. wake bout lengths for different levels of LC connectivity for N = 500. Note the y-axis is consistent for all cases, but the x-axis differs between sleep and wake. **Left (SA case):** Increasing the LC strength does not change the average sleep bout length much, and the distribution remains exponential. **Right: (WA case):** Wake bout durations lengthen on average as LC strength increases, and the shape of the distribution of bout lengths differs noticeably from exponential in the case of no LC interaction. In panel b), the inset graph shows the same data, but plotted with the same x-axis as the SA case. This shows that with no LC interaction, the WA and SA distributions are nearly identical. Larger x-axis is used for WA plots to show the increase in observed bout lengths as LC-WA interaction is increased.

Fig 15 shows the distribution of sleep and wake bouts in the different cases of LC strength which corresponds to different levels of mammalian maturity: infant (no LC), adolescent (moderate LC strength), adult (strong LC strength). We see similar behavior as in the N = 5 case. As LC strength increases, we get longer wake bouts whereas sleep bout lengths remain fairly consistent. Compare Fig 15 (large network) to Fig 11 (small network).

**Fig 16:**
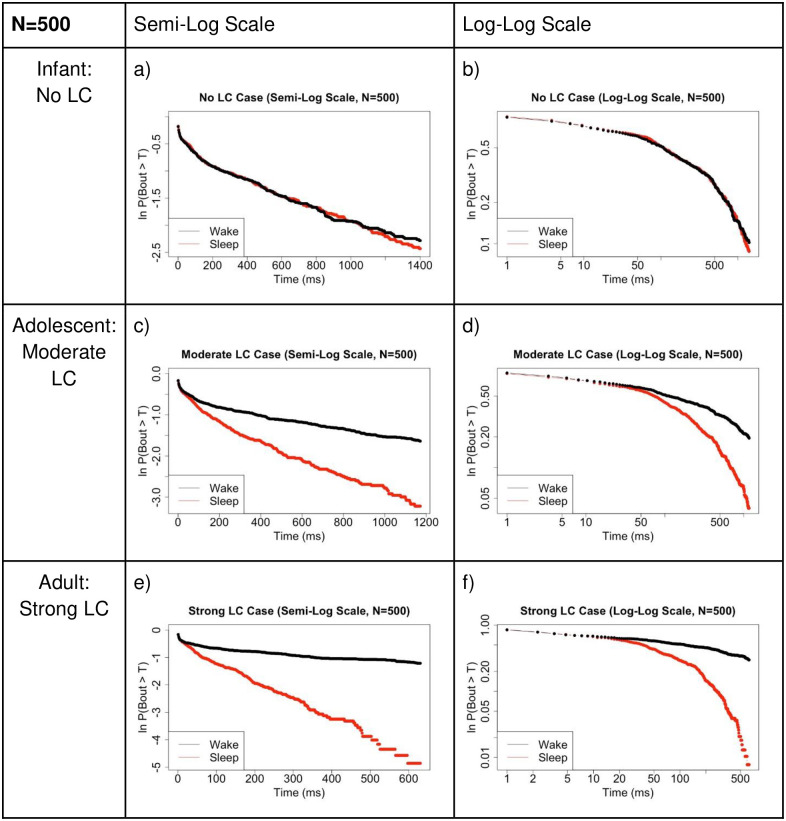
Larger network comparison of survivor plots. Comparison of survivor plots of wake and sleep bout distributions as connectivity strength between the WA population and LC population is increased in multiple ways, N = 500 case. **Left:** Semi-log scale; **Right:** Log-log scale. We observe the predicted shift in wake bout distribution from exponential to power law-like once the model brain has reached ‘maturity’. Compare to Fig 12 which shows identical plots for the N = 5 case. We see maintenance of the network behavior at this much larger neuron population size. Refer to Fig 4 which shows that a power law distribution is linear on a log-log scale and an exponential distribution is linear on a semi-log scale. These data were obtained from 100,000 time step simulations.

We also regenerate semi-log and log-log plots in Fig 16 for the large network case (N = 500) similar to Fig 12 in the small network case (N = 5). Importantly, we see very similar behavior in this comparison as well. In the No LC case, the wake and sleep bouts appear nearly identical. As LC strength is increased, representing maturation of the mammal, we see the formation of a linear appearing log-log plot for the wake bouts, in accordance with the prediction of a transition to a power law-like distribution.

**Fig 17:**
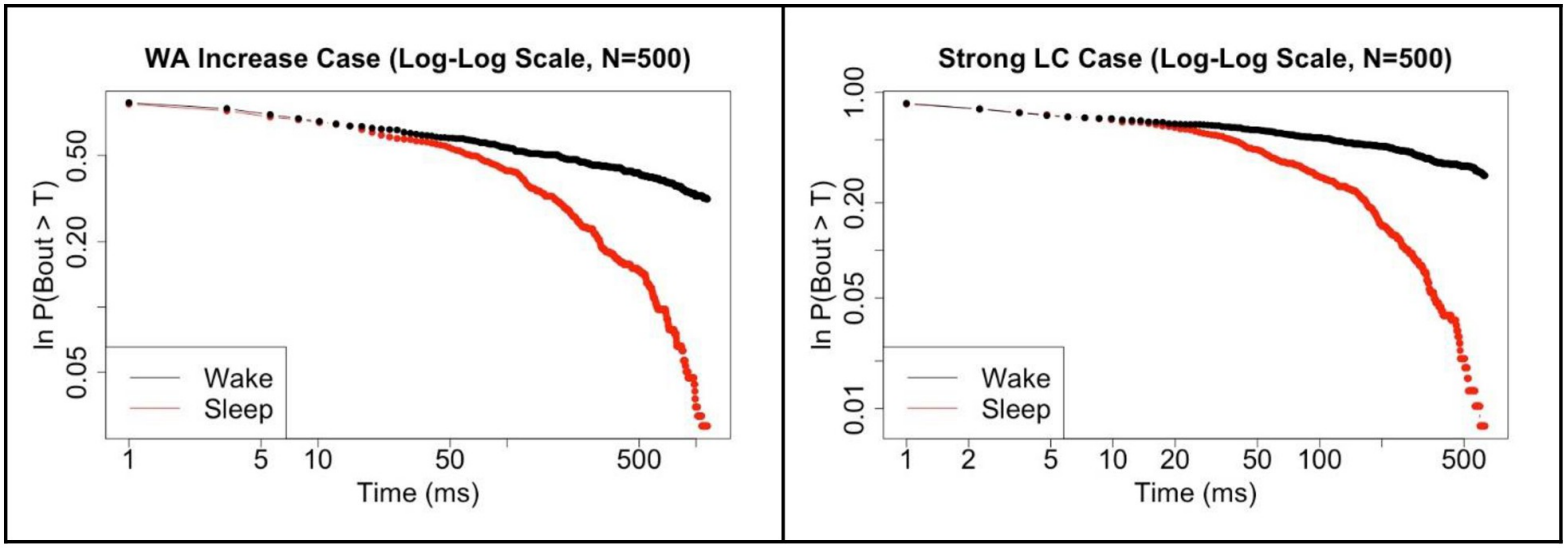
Strong wake-active self-excitation vs strong wake-promoting LC connectivity. Comparison of survivor plots of wake and sleep bout distributions for the No LC case with increased WA self-excitation (**left**) versus the Strong LC case (**right**) for N = 500.

Fig 17 shows one last comparison which addresses the question: Is there a difference between WA-WP population interactions and simply a WA neuron population with very strong self-excitation? We see somewhat similar results for the parameter values used, but further work here would be needed to fully answer this question.

Our investigation supports the three-neuron population model as a more realistic representation of neuronal connectivity related to sleep-wake behavior in the developing mammal.

## Discussion

We expanded upon previous work [18–20] by developing a novel integrate-and-fire neuronal network model to emulate observed sleep-wake behavior throughout mammalian development. This included three different neuron populations: wake-active (WA), sleep-active (SA), and wake-promoting (WP), which correspond to the dorsolateral pontine tegmentum, nucleus pontis oralis, and locus coeruleus (LC), respectively. This model expands our understanding of neuronal networks and mechanisms in the mammalian brain responsible for sleep-wake behavior throughout development. In particular, we confirm the notion that the wake-promoting brain region (LC) contributes to the increase in average wake bout length, as well as the transition of wake bout distribution from exponential to power law. This model is more sophisticated than previous models as it incorporates a more flexible and complex framework by incorporating intrapopulation neuronal network structure, as well as a more realistic representation of external noisy stimuli to each individual neuron in the system. The stochasticity of the system comes from external excitatory noise; our model integrates independent noise at each time step for each individual neuron, instead of spreading one value evenly over the entire population, as in the Wilson-Cowan style model.

Additionally, our model allows for a larger neuron population size and finer control over synaptic connectivity strength and other parameters regarding neuronal connectivity, as compared to previous models. We delve deeper into quantitative analysis of the interaction between LC-WA connectivity, the implications of this connectivity within an individual neuron population, and the effect of this relationship on gross network behavior. We investigate how both inter and intrapopulation neuronal network structure and connectivity strength contribute to the observed sleep-wake behavior of the model. This dynamic neuronal connectivity is a possible mechanism that accounts for sleep-wake pattern changes observed during mammalian development.

With the inclusion of neuronal network structure and connectivity, our first goal was to confirm that previously established network behavior patterns had not been disrupted. Through the implementation of similar SA-WA and WA-SA mutual-inhibition, WA, SA and LC self-excitation, and noisy excitatory external stimuli, we observed the overall preservation of switching behavior necessary to model sleep and wakefulness. This bistable stochastic system is the framework on which the rest of the model is built. We observe, similar to previous models, that the mutual inhibition between the WA and SA populations must be large in comparison to the individual population self-excitation, and the noisy external stimuli is required to induce switching between the two bistable states.

Likewise, we confirmed the previous observation that mean wake and sleep bout lengths can be independently controlled solely by the manipulation of the strength of mutual-inhibition synaptic connectivity. This is an important property of the model to be realistic in replicating behaving rat data, as one of the crucial findings is that both sleep and wake bout lengths do increase over time through mammalian development. This factor can be accounted for by a gradual increase in mutual inhibition between SA and WA neuron populations. The goal of this work, however, is primarily to account for the underlying network structure that results in sleep bouts maintaining an exponential distribution while wake bouts transition to a power law distribution during mammalian development.

By simultaneously tracking the activity of the three modeled neuron populations, we see strong correlation between WA and WP (LC) activity due to the increasingly strong interaction between the two populations. This strong co-population activity likely contributes to the observed changes in the network behavior. Stronger neuronal connectivity between the WA and LC populations would lead to higher levels of correlative firing behavior. To inspect this relationship quantitatively, we measured the LC firing rate during both sleep and wake bouts. We found that in the N = 5 case, the LC brain region displayed about 2.7 times more activity during a wake bout than a sleep bout.

To dive a bit deeper into the mechanisms by which the network behavior is being produced, we tracked the voltage (membrane potential) of representative neurons within each population over time (most importantly SA and WA). We see that a period of activity for a certain population results in membrane depolarization (above RMP). Even more significantly though, we see that the inactive population is driven to hyperpolarization. These are direct consequences of the neuronal connectivity. The WA and SA populations both exhibit self-excitation, leading to perpetuated activity within the already active population. The SA and WA mutual inhibition drives the inactive population further into hyperpolarization, making it even more unlikely/difficult for it to become active, contributing to the bistable states of the model. This process is exacerbated by the inclusion of the WP brain region, which adds even further excitation to the WA population during a wake bout. Now that we understand how the population-level network connectivity may be contributing to overall network behavior, we compared the distribution of sleep and wake bouts in multiple ways to see if this network structure could account for the findings of observed rat data.

In the histograms of the sleep versus wake bout lengths, we see that the SA bout distribution maintains not only a similar shape, but also a nearly identical mean bout length as the LC and WA interaction (synaptic connectivity) is strengthened. This is what we predict based on observed data, which supports this model and neuronal connectivity as representative of reality. For WA bouts, we see that with no LC-WA connectivity (representing mammalian infanthood), WA and SA bout distributions appear nearly identically with similar mean bout length. This makes sense, as during infanthood, both SA and WA bouts should be exponentially distributed. As LC and WA interaction is strengthened, we see that WA bout lengths have a more heavy-tailed distribution with significantly longer maximum and mean bout lengths. This is consistent with the transition of WA bouts from an exponential to a power law distribution as the rat reaches adulthood.

We next plotted this same data on semi-log and log-log plots to confirm which distributions are present. In the no LC case, both populations are linear on a semi-log plot, indicating an exponential distribution of WA and SA bouts during infanthood. As LC and WA synaptic connectivity is increased, the SA bout distribution maintains a nearly identical shape on the semi-log plot, lending to the maintenance of an exponential distribution in the SA bout lengths. Finally, and importantly, we note that as LC and WA synaptic connectivity strength increases, the WA bouts become increasingly linear on a log-log plot, which is indicative of a shift towards a power law-like distribution during rat adulthood. This modeled change in synaptic connectivity between the three brain regions accurately accounts for the observed changes in mammalian sleep-wake bout duration data through development.

After working extensively with the N = 5 case, we expanded the size of our network model to N = 50 and N = 500 for each neuron population. This not only shows the flexibility of our model, but also gives insight into the behavior of the network at a larger, more realistic neuron count. Our model necessitated changes to the synaptic strengths between and within neuron populations to produce similar behavior, see Table 4. We modeled all three cases of LC synaptic strength in a similar fashion and found very similar results. We see that as LC strength is increased, we have longer and longer wake bouts compared to sleep bouts. When analyzing the semi-log and log-log plots, we observe that this emulates a change from an exponential distribution to a power law-like distribution in the wake bout lengths as the rat matures into adulthood.

During rat adolescence to adulthood (P8-P21), the LC neuronal network changes its oscillatory properties from low frequency / large amplitude at P8 to increased frequency / decreased amplitude, with oscillations and synchrony disappearing at P21. We do not include these features in our LC network, but we are still able to reproduce key features of this system with a simpler LC population. Our model supports the idea that the change in interaction between the WA and LC populations as the rat matures is due to multi-way increased synaptic connectivity: WA → LC, LC → WA, SA → LC, and LC → LC. We find that this has similar effects as increasing the frequency of interaction between LC and WA. This stronger positive feedback loop leads to more LC spikes and thus more spikes in the WA population.

Future work could expand on many aspects of our neuronal network model. Recall that we used all-to-all connectivity in our model due to the relatively small numbers of neurons modeled in each population, but future work could investigate to what degree intrapopulation connectivity affects neuronal network performance and function. One could easily alter only one interpopulation neuronal connectivity strength at a time and see how this would contribute to overall network behavior, specifically if this would still recreate the desired sleep-wake bout distribution shift. Similarly, additional brain regions could be added, as it is likely that in a fully developed mammalian brain, there are multiple regions which are active during wakefulness and multiple regions active during sleep. Finally, further study and analysis of the N = 500 case could be beneficial to understand more about the network behavior on this larger scale.
